# Impact of non-ignorable missingness on genetic tests of linkage and/or association using case-parent trios

**DOI:** 10.1186/1471-2156-6-S1-S90

**Published:** 2005-12-30

**Authors:** Chao-Yu Guo, Jing Cui, L Adrienne Cupples

**Affiliations:** 1 Department of Mathematics and Statistics, Boston University, Boston, MA, USA; 2 Department of Medicine, Boston University, Boston, MA, USA; 3 Department of Biostatistics, Boston University, School of Public Health, Boston, MA, USA; 4 NHLBI's Framingham Heart Study, 111 Cummington Street, Framingham, MA 02215, USA

## Abstract

The transmission/disequilibrium test was introduced to test for linkage disequilibrium between a marker and a putative disease locus using case-parent trios. However, parental genotypes may be incomplete in such a study. When parental information is non-randomly missing, due, for example, to death from the disease under study, the impact on type I error and power under dominant and recessive disease models has been reported. In this paper, we examine non-ignorable missingness by assigning missing values to the genotypes of affected parents. We used unrelated case-parent trios in the Genetic Analysis Workshop 14 simulated data for the Danacaa population. Our computer simulations revealed that the type I error of these tests using incomplete trios was not inflated over the nominal level under either recessive or dominant disease models. However, the power of these tests appears to be inflated over the complete information case due to an excess of heterozygous parents in dyads.

## Background

When parental genotypes are missing at random (MAR) in case-parent trio studies, Clayton [1] and Weinberg [2] suggested a partial-score test and a likelihood ratio test, respectively, to deal with such data. Under the same situation, Sun et al. [3] introduced the 1-TDT, a transmission/disequilibrium test (TDT)-type test based on a set of non-iterative estimates of the genotype relative risk (GRR) [4]. Recently, the expectation maximization-haplotype relative risk (EM-HRR) proposed by Guo et al. [5] extended the HRR test [6] to accommodate trios with one or no parental genotypes, and it outperforms the 1-TDT in a homogeneous population. However, when the MAR assumption is violated, occurring when missingness is non-ignorable due, for example, a missing pattern of parental genotypes is related to the disease under study, these tests may be invalid.

To assure a valid test for association between a marker and a putative disease locus under non-ignorable missingness (NIM), Allen et al. [7] introduced a testing procedure based on the joint likelihood of the genotypes of the proband and the observed parents, conditioning on the proband's phenotype and parental missingness pattern. Still, the validity of their method under population stratification is not guaranteed, because it depends on whether the missingness model is suitably specified or not. Therefore, Chen [8] proposed another TDT-type approach based on the conditional likelihood of the proband's genotype given the number and, if any, genotypes of available parents, as well as the proband's phenotype to assure the validity of testing for association between a candidate gene and a disease.

The cost of accounting for NIM is a loss of power under MAR (it is less powerful than the 1-TDT) as indicated by Allen et al. [7]. Their results also suggested that, under NIM, the 1-TDT performs better than the proposed tests by Clayton [1] and Weinberg [2], because the type I error of the 1-TDT is less inflated over the nominal level. In addition, the 1-TDT is a valid test if the NIM is a result of population stratification, while Clayton [1] and Weinberg's [2] methods are not. Hence, the 1-TDT is preferred among those tests for incomplete trios that require the MAR assumption. Because the comparison between the 1-TDT and EM-HRR under NIM is unknown, we examined the performance of the two tests using Genetic Analysis Workshop 14 (GAW14) simulated data.

## Methods

### EM-HRR

First consider a diallelic marker with alleles B_1 _and B_2_.  represent the observed count for each type of trio data, where k = 0, 1, or 2 represents total number of B_1 _alleles transmitted to the offspring, and i, j = 0, 1, or 2 represents total number of B_1 _alleles for fathers and mothers, respectively. Note that the superscript * is used when the parental genotype is missing. Curtis and Sham [9] showed that bias in estimating the probability of transmission of certain alleles is introduced if heterozygous affected children with one heterozygous parent families are excluded. For simplicity we denote these dyad families by  whenever the father or mother is missing, because we assume no difference according the sex of the parent. Guo et al. [5] applied the EM algorithm to estimate the proportion of heterozygous parents transmitting B_1 _and not B_2 _() and transmitting B_2 _and not B_1 _() alleles among  families to avoid such bias. The details of the EM procedure are available in Guo et al. [5].

The HRR compares parental marker alleles transmitted to an affected child to those not transmitted. One feature of the HRR for dealing with such trio-type family data is that the affected children's genotypes are always known (assuming no genotyping failure) due to ascertainment procedures in which data from an affected individual is collected first and then that of his/her parents. Hence, in the case group, the two transmitted alleles of all affected children are known and can be used in the analysis, even when both parents' genotypes are not available.

Let U_i_, V_i_, W_i_, and X_i _represent the total number of transmitted B_1 _alleles, non-transmitted B_1 _alleles, transmitted B_2 _alleles, and non-transmitted B_2 _alleles from type i families, where i = 1 for complete trios, 2 for dyads (trios with one parental genotype available), and 3 for monads (trios without parental genotypes). Note that only  and  require the EM estimates; the rest can be inferred without the EM algorithm and can be uniquely determined. Both V_3 _and X_3 _are 0, because no parental genotypes are available to infer what alleles are not transmitted.

The EM-HRR is defined as (U_1 _+ U_2_) × (X_1 _+ X_2_) / (V_1 _+ V_2_) × (W_1 _+ W_2_), if type 3 families are excluded. If all families ascertained are used for analysis regardless of missing one or two parents, then the EM-HRR becomes (U_1 _+ U_2 _+ U_3_) × (X_1 _+ X_2_) / (V_1 _+ V_2_) × (W_1 _+ W_2 _+ W_3_). Under the null hypothesis of no linkage or no association, the EM-HRR is expected to be 1 and the test statistic  follows a central chi-square distribution with 1 degree of freedom. Note that *Var*(*EM *- *HRR*) can be approximated by (), if the type 3 families are excluded and by (), if all three type of families are used.

### Simulations

One affected child was randomly selected in each nuclear family in order to maintain the independence among ascertained trios. Both dominant and recessive disease models were examined, since we used traits "b" (dominant) and "l" (recessive) for ascertainment. For trait b, SNPs C01R0052 and C01R0001 were used in power and type I error simulations, respectively. Similarly, SNPs C09R0765 and C09R0850 were used for trait l (several loci were examined with similar results but not shown here). Based on resampling of the 100 replicates provided, 10 replicates in the Danacaa (DA) population were randomly selected for each simulation. A total of 1,000 simulations were conducted for power and type I error comparisons.

The TDT and HRR were first applied to the complete trios. To illustrate the impact of NIM, we examined the extreme case by assigning parental genotypes to be missing if they were affected. We first determined the missing rate for parents in the NIM simulations (there was no difference in sex specific missing rates), then generated a MAR dataset of equal amounts of missing data by randomly assign parental genotypes to be missing according to that rate. The 1-TDT and EM-HRR were both applied to the subset of complete trios and dyads, but only EM-HRR can accommodate monads under NIM and MAR.

## Results

The average sample sizes of ascertained trios are 120 and 750 for the recessive trait l and dominant trait b, respectively. The average missing rates for each parental genotype are approximately 10% and 30% for recessive trait l and dominant trait b, respectively. In Tables 1 and 2, the rows marked TDT_trad _and HRR_trad _present the results for the traditional tests using all unrelated trios. TDT_comp _and HRR_comp _tests used the traditional TDT and HRR tests on the subset of complete trios only after assigning parental genotypes to be missing. 1-TDT and EM-HRR_dyads _tests used both subsets of complete trios and dyads. EM-HRR_all _used three types of trios.

As in results reported by Ewens et al [11], the TDT and HRR perform similarly (comparable power in TDT_trad _and HRR_trad_, or TDT_comp _and HRR_comp_) in detecting linkage disequilibrium (LD) between a marker and a putative disease locus in a homogeneous population. In all the situations simulated, TDT_comp _and HRR_comp _have the lowest power, and the difference between TDT_trad _and TDT_comp _or HRR_trad _and HRR_comp _is the loss of power due to exclusion of incomplete trios. The increase from TDT_comp _to 1-TDT or HRR_comp _to EM-HRR_dyads _represents a gain of power by including dyads. The difference between EM-HRR_dyads _and EM-HRR_all _indicates the gain or loss of power by additionally utilizing monads, which is not applicable for the 1-TDT test. Because the transmitted alleles are always present regardless of missing one or two parental genotypes in the HRR statistic, EM-HRR_dyads _and EM-HRR_all _are more powerful than 1-TDT under both dominant and recessive disease models regardless of MAR or NIM.

Under NIM, the probability distribution functions of monads changed the most compared to dyads, which resulted in adding more noise to the EM-HRR statistic. As a consequence, we observed a loss of power in the EM-HRR due to the utilization of monads. Therefore, EM-HRR_all _is more powerful than EM-HRR_dyads _under MAR, but their performances are reversed when the missing pattern is informative.

When parental genotypes are missing non-randomly due to a recessive disease, only homozygous parents with two copies of the disease alleles will be missing, assuming there are no phenocopies. Therefore, the subset of complete trios or dyads has more heterozygous (informative) parents compared to those under MAR. One can see that, under NIM, the loss of power from TDT_trad _to TDT_comp _or HRR_trad _to HRR_comp _is less compared to the MAR situation. In addition, the EM procedure yields higher informative transmissions based on excess heterozygous (informative) parents. Therefore, the power of EM-HRR using both the subset of complete trios and dyads is higher than HRR using the complete dataset (Table 1). The results are similar under a dominant disease model as seen in Table 2.

Allen et al. [7] and Chen [8] showed that type I errors of MAR tests were inflated over the nominal level. Although our simulations results did not match theirs, we see informative changes in the type I errors. When parental genotypes are missing non-randomly due to a dominant disease, parents with two copies of normal alleles are not affected, assuming no phenocopies. Hence, these types of parents will be more likely to be in the subset of complete trios and dyads. It is evident then that the loss of power from TDT_trad _to TDT_comp _or HRR_trad _to HRR_comp _is greater compared to the loss under a recessive disease model. This phenomenon also affects the type I error. Hence the type I error of HRR_comp _and TDT_comp _are smaller than TDT_trad _and HRR_trad _(Table 2), but for a recessive disease, the results are reversed (Table 1), because the heterozygous parents are more likely to be in the subset of complete trios and dyads.

## Conclusion

The HRR was the first family-based test for LD between a marker and a putative disease locus. Because the TDT performs better than the HRR under extreme admixture, the HRR is not as popular as the TDT. Due to the data structure of the HRR, the transmitted alleles are always present regardless of the absence of one or both parents. Therefore, the EM-HRR is more powerful than the 1-TDT when the population is under Hardy-Weinberg equilibrium or slightly admixed. Because there is no admixture in the DA population, we found that the EM-HRR is the more powerful test when parental genotypes are missing randomly, and the superiority of the HRR remains despite the impact of NIM. Because the use of affected children without parental genotypes does not improve the power of the EM-HRR with NIM, we recommend using the EM-HRR with the subsets of complete trios and one-parent data for testing LD between a marker and a putative disease locus when the missing data pattern is unknown.

Under a different mechanism of NIM and no phenocopies in the simulated Dananca population, our results do not match those of the 1-TDT with inflated type I error reported by Allen et al. [7] and Chen [8]. Instead, we observed a different performance of MAR tests under NIM. Although it is easier to observe that the 1-TDT and EM-HRR_dyads _are more powerful than TDT_trad _and HRR_trad _when their type I errors are inflated over the nominal level, our results suggest that in the GAW14 simulated data, parents with different genotypes are equally likely to be diseased under the null hypothesis, and that differential missing rates occur only under the alternative hypothesis.

## Abbreviations

EM: Expectation maximization

GAW14: Genetic Analysis Workshop 14

GRR: Genotype relative risk

HRR: Haplotype relative risk

LD: Linkage disequilibrium

MAR: Missing at random

NIM: Non-ignorable missingness

TDT: Transmission/disequilibrium test

## Figures and Tables

**Table 1:** Recessive trait (L): 120 trios on average

	Locus: C09R0765		Locus: C09R0850	
	Power %		Type I error %	
		
	NIM*	MAR**	NIM	MAR
TDT_trad_	34.2		3.1	
HRR_trad_	34.2		3.0	
TDT_comp_	33.7	27.1	4.7	3.1
HRR_comp_	33.6	27.1	5.4	3.0
1-TDT	39.5	31.7	4.5	2.9
EM-HRR_dyads_	44.2	31.9	3.9	3.1
EM-HRR_all_	42.3	32.1	3.3	3.3

* Missing rate for each parent is approximately 10%.

** Each parent is missing randomly at the same rate of NIM.

**Table 2:** Dominant Trait (B): 750 trios on averge

	Locus: C01R0052		Locus: C01R0001	
	Power %		Type I error %	
		
	NIM*	MAR**	NIM	MAR
TDT_trad_	16.2		5.5	
HRR_trad_	16.4		5.3	
TDT_comp_	11.5	9.8	4.6	4.7
HRR_comp_	11.4	10.0	4.4	4.6
1-TDT	20.4	12.5	5.1	4.8
EM-HRR_dyads_	23.5	14.1	4.3	4.4
EM-HRR_all_	22.8	14.2	4.4	4.6

* Missing rate for each parent is approximately 30%.

** Each parent is missing randomly at the same rate of NIM.
